# Targeting Adult Mesenchymal Stem Cells Plasticity for Tissue Regeneration

**DOI:** 10.1155/2017/4532179

**Published:** 2017-06-21

**Authors:** Giorgio Mori, Giacomina Brunetti, Filiberto Mastrangelo, Elisabetta A. Cavalcanti-Adam

**Affiliations:** ^1^Department of Clinical and Experimental Medicine, University of Foggia Medical School, Via L. Pinto, 71122 Foggia, Italy; ^2^Department of Basic Medical Sciences, Neuroscience and Sense Organs, Section of Human Anatomy and Histology, University of Bari “A. Moro”, Piazza Giulio Cesare 11, 70124 Bari, Italy; ^3^Institute of Physical Chemistry, Department of Biophysical Chemistry, University of Heidelberg and Max Planck Institute for Intelligent Systems, Stuttgart, Germany

Tissue damage derived from tumors, traumatic events, inflammatory diseases, or just aging causes life quality impairment. Thus, tissue regeneration not only represents the main goal of regenerative therapy but also is certainly one of the greatest challenges of modern medicine.

Mesenchymal stem cells (MSCs) can be isolated from different source tissues in the adult organism. MSCs can differentiate in mature cells of different lineages; thus, if opportunely targeted, they have the potential to regenerate and heal the injured tissue. The scientific contributions which are part of this special issue present and analyzed these items both in the form of research articles and reviews.

The bone marrow is still the gold standard tissue for harvesting MSCs: different clonal cells were analyzed showing heterogeneity; alkaline phosphatase assay could indicate the precursor's lineage (M. Elsafadi et al.). Dental tissues also contain MSCs: in particular, the dental follicle [1] and dental bud [2] are striking sources of cells which readily differentiate into the osteoblastic lineage. Dental pulp stem cells successfully undergo osteogenic differentiation [3] and their reparative behavior could be increased by platelet lysate supplementation (P. Marrazzo et al.). For bone tissue regeneration and vascularized bone grafts, both adipose tissue and periodontal ligament stem cells could be differentiated into bone cells, and in particular, adipose cells can be successfully cryopreserved for tissue banking (I. Roato et al.).

A great challenge in regenerative therapy is the guidance of wound healing. MSCs promote wound healing, and human umbilical cord MSCs accelerate wound repair and hair follicle regeneration via Wnt overexpression (L. Dong et al.). Interestingly, as shown by X. Liu et al., overexpression of semaphorin 3A in adipose-derived stem cells leads to a phenotype switch toward the osteoblastic lineage by upregulating the Wnt pathway as well. These studies on alternative sources of MSCs highlight the importance of molecular signals that might direct MSC fate and more specifically guide them to form mineralized tissue [4].

To increase healing at bone fracture sites, several approaches that stimulate physically the differentiation of MSC have been developed. Pulsed electromagnetic fields promote bone marrow MSCs osteogenic differentiation by activating TGF-*β* signaling pathway and stimulating the expression of microRNA21 (N. Selvamurugan et al.). Given that natural composition and structure of the extracellular environment has a profound impact on MSC osteogenic differentiation [5], a variety of scaffolds and matrix-based materials are used in dentistry and craniofacial surgery. Combining both the priming MSC with stem cell chemical modulators and the use of 3D printing-based materials represents a successful strategy not only for the treatment of cardiovascular disease but also for fostering revascularization of grafts in a variety of tissues.

*Giorgio Mori* *Giorgio Mori* *Giacomina Brunetti* *Giacomina Brunetti* *Filiberto Mastrangelo* *Filiberto Mastrangelo* *Elisabetta A. Cavalcanti-Adam* *Elisabetta A. Cavalcanti-Adam*
